# Dynamic changes in autophagy activity in different degrees of pulmonary fibrosis in mice

**DOI:** 10.1515/biol-2022-0860

**Published:** 2024-05-28

**Authors:** Xiulan Chen, Xin Lin, Lihuan Xu, Yu Liu, Xin Liu, Chunhui Zhang, Baosong Xie

**Affiliations:** Department of Clinical Medicine, Fujian Medical University, Fuzhou, Fujian 350004, China; Department of Respiratory and Critical Care Medicine, Fujian Provincial Geriatric Hospital, Fuzhou, Fujian 350009, China; Department of Respiratory Medicine, Mengchao Hepatobiliary Hospital of Fujian Medical University, Fuzhou, Fujian 350025, China; Department of Internal Medicine, Fujian Provincial Hospital, Fuzhou, Fujian 350013, China; Department of Respiratory and Critical Care Medicine, Fujian Provincial Hospital, No. 134 East Street, Fuzhou, Fujian 350013, China

**Keywords:** pulmonary fibrosis, autophagy activity, bleomycin

## Abstract

The aim of this study is to observe the changes in autophagy activities in lung tissues of mice with different degrees of pulmonary fibrosis (PF), and explore the association between PF and autophagy activity. The PF model was established by bleomycin (BLM, 25 and 35 mg/kg) atomization inhalation in C57BL/6 mice, samples were collected on the 7, 14, and 28 days after BLM administration. Hematoxylin–eosin staining was used to observe the pathological changes in lung tissues. Masson staining was utilized to assess areas of blue collagen fiber deposition in lung tissues. Quantitative real time polymerase chain reaction was used to detect the mRNA expressions of autophagy-related genes, including Atg5, Atg7, and Atg10 in lung tissues. Western blot was used to detect the protein expressions of autophagy-related genes, including p62 and LC3II/LC3I in lung tissues. Compared with control group, BLM dose-dependently decreased PaO_2_, mRNA expressions of Atg5, Atg7, Atg10, and LC3II/LC3I, while increased lung wet weight, lung coefficient, PF score, the blue area of collagen fibers, and p62 protein on the 7th, 14th, and 28th days. In conclusion, the more severe the PF induced by BLM, the lower the autophagy activity.

## 1 Introduction

Idiopathic pulmonary fibrosis (IPF) is a progressive lung disease which occurs owing to the aberrant fibrosis response to chronic epithelial cell injury [1]. IPF is incurable with an average onset at about 62 years old, of which 54% are male [2]. IPF affects approximately 0.9–13 per 100,000 individuals globally with the rising incidence year by year [3]. IPF is characterized by the nonproductive cough and progressive dyspnea [4]. In IPF, the elevated extracellular matrix substitutes for healthy lung tissues and disrupts alveolar architecture, thus destroying pulmonary compliance, leading to respiratory failure and death [5]. IPF might be caused by a combination of genetic and environmental factors; however, the etiology of IPF remains unknown [6]. The risk factor of IPF, e.g., tobacco exposure is implicated, the history of cigarette smoking is frequent [7]. For patients with advanced IPF, the only recommended and effective therapy is lung transplantation [8]. Consequently, it demands aggressive pursuit of better choices for curative therapy of IPF.

Recently, lack of autophagy as a potential pathogenesis of IPF has been paid more and more attention, the majority of studies propose that reduced autophagy activity aggravates IPF [9,10]. Previous findings of our lab also confirmed this phenomenon [11].

In the present study, it is found that different doses of BLM induced different severities of pulmonary fibrosis (PF) in mice. The more severe the PF lesion, the more obvious the decline in autophagy activity.

## 2 Materials and methods

### 2.1 Reagents

Bleomycin (Invitrogen, San Diego, USA), Masson staining Kit (Fuzhou Maixin Biotechnology Development Company, Fuzhou, China), diaminobenzidine chromogenic Kit (Fuzhou Maixin Biotechnology Development Company, Fuzhou, China), RNA extract (Wuhan Servicebio Technology, Wuhan, China), First Strand cDNA Synthesis Kit (Takara, Osaka, Japan), fluorescence quantitative PCR instrument (7300, Applied Biosystems, Foster, USA), RIPA lysate (Solarbio, Beijing, China), phenylmethanesulfonylfluoride (PMSF) and protease inhibitor mixture (Roche Diagnostics, Indianapolis, USA), BCA protein Quantification Kit (Thermo Fisher Scientific, Waltham, USA), P62 and LC3II/I antibody (Cell Signaling Technology, Boston, USA), horseradish peroxidase (HRP) goat anti-rabbit antibody (Wuhan Servicebio Technology, Wuhan, China), ECL luminescent solution (Solarbio, Beijing, China), blood gas analyzer (Abbott, Shanghai, China), Masson’s staining kit (Solarbio, Beijing, China), NIKON Eclipse 80I optical microscope (Nikon, Tokyo, Japan), NIKON Eclipse TE2000-U inverted microscope (Nikon, Tokyo, Japan), table top high-speed cryogenic microcentrifuge (D3024R, DLAB, Shanghai, China), and microtome (Leica Microsystems, Solms, Germany) were procured.

### 2.2 Experimental animals

In total, 72 healthy male SFP C57BL/6 mice, 6–8 weeks old, weighing 17.0–19.0 g, were purchased from Shanghai Slyke Laboratory Animal Company (Shanghai, China). Mice were kept in a specific pathogen-free barrier system, with controlled room temperature of 23 ± 2°C, photoperiod of 12 h light-dark cycle, and humidity of 60%.

Mice were allowed to acclimate to the new environment for a week, then they were randomly divided into control (ctrl, *n* = 8), BLM low (25 mg/kg, *n* = 8), and high (35 mg/kg, *n* = 8) groups. Four mice of the same group with the same weight as close as possible were put into a self-made transparent acrylic box (20 cm × 23 cm × 18 cm) each time, followed by connection to the atomization device, with the atomization time of 40 min. Mice in ctrl group were given aerosol inhalation of saline, while mice in BLM groups were given aerosol inhalation of 25 and 35 mg/kg of BLM, respectively. Samples were collected on the 7th, 14th, and 28th days after BLM administration, as referred to a previous report [12].


**Ethical approval:** The research related to animal use has been complied with all the relevant national regulations and institutional policies for the care and use of animals, and has been approved by the Animal Ethics Committee of Fujian Medical University.

### 2.3 Abdominal aortic blood gas analysis

Mice were subjected to preoperative fasting and water deprivation for 12 h, then anesthetized by intraperitoneal injection of 2% sodium pentobarbital (50 mg/kg). The procedures were as listed: cut the skin from the pubic bone along the ventral midline to the xiphoid process, open the peritoneum to fully expose the abdominal cavity, locate the abdomen behind the abdominal aorta and peel off the tissue surrounding the blood vessels, puncture the abdominal aorta towards the proximal end with a blood collection needle to extract 0.1–0.2 mL of arterial blood, and immediately subject it to arterial blood gas analysis.

### 2.4 Lung coefficient

The body weight of mice in each group were measured. Accordingly, mice were sacrificed by abdominal aortic bloodletting, the lung tissues were removed, and the wet weight of both lungs was measured. Lung coefficient was calculated with the following equation: lung coefficient = wet lung weight/mouse weight.

### 2.5 Hematoxylin–eosin (HE) staining

HE staining was conducted to evaluate the pathological changes in lung tissue and lung fiber score, as referred to a previous report [13]. In brief, lung tissues were fixed by 4% polyformaldehyde (PFA) at 4°C overnight, embedded in paraffin, sectioned into 4 μm slices by a microtome, and incubated in an oven of 60°C for 30 min. Next slices were dewaxed by xylene I (10 min) and xylene II (10 min). Then, slices were hydrated by absolute ethanol (5 min), 95% ethanol (2 min), 85% ethanol (2 min), and 75% ethanol (2 min). Subsequently, slices were immersed in 10% hematoxylin (5 min), differentiated by 75% ethanol hydrochloride (15 s), and stained by 0.5% eosin (3 min). At last, slices were dehydrated by 95% ethanol (10 min) and absolute ethanol (10 min), permeabilized by xylene I (10 min) and xylene II (10 min). Five fields were randomly selected under a light microscope (×200). Each successive field was individually assessed for the severity of fibrosis in HE-stained lungs and quantified in a blinded fashion by two pathologists using an Ashcroft scoring system [14]. Image Pro-Plus 6.0 was applied for data analysis.

### 2.6 Masson staining

Masson staining kit was utilized to assess collagen fiber deposition in lung tissues of mice. Briefly, lung tissues were fixed by 4% PFA at 4°C overnight, embedded in paraffin, sectioned into 4 μm slices using a microtome, and incubated in an oven at 60°C for 30 min. Next slices were dewaxed by xylene I (10 min) and xylene II (10 min). Then, slices were hydrated by absolute ethanol (5 min), 95% ethanol (2 min), 85% ethanol (2 min), and 75% ethanol (2 min). Subsequently, slices were immersed in 10% hematoxylin (5 min), differentiated by 75% ethanol hydrochloride (15 s), and stained by Masson staining solution (5 min), Ponceau 2R (5 min), and aniline blue staining solution (2 min). At last, slices were dehydrated by 95% ethanol (10 min) and absolute ethanol (10 min), permeabilized by xylene I (10 min) and xylene II (10 min). Five fields were randomly selected under a light microscope (×200). Image Pro-Plus 6.0 was used to measure and calculate the areas of blue collagen fiber deposition.

### 2.7 Quantitative real time polymerase chain reaction (qRT-PCR)

qRT-PCR was carried out to evaluate the expression of mRNAs, as per a previously described study [15]. Total RNA was extracted from lung tissues by Trizol. RNA concentration and purity were detected by Nanodrop 2000 spectrophotometer. Then, mRNA was reverse transcribed into cDNA by First Strand cDNA Synthesis Kit. qRT-PCR was performed on 7300 Fast Real-Time system. Thermocycle conditions were: 95°C for 10 min, 35 cycles at 95°C for 20 s, and 58°C for 1 min. Relative mRNA expressions of Atg5, Atg7, and Atg10 were quantified by 2^–ΔΔCT^ method. GAPDH acted as an endogenous control. Primer sequences are shown in Table 1.

**Table 1:** Primer sequences

Gene	Forward (5′−3′)	Reverse (5′−3′)
Atg5	TATCAGACCACGACGGAGCG	GGTTTCCAGCATTGGCTCTATC
Atg7	GTTTCCAGTCCGTTGAAGTCCT	GCCTCCTTTCTGGTTCTTCTCC
Atg10	GAATGGAGAACAGCCAAGGAAT	AACGGTCTCCCATCTAAAAAGC
GAPDH	CCTCGTCCCGTAGACAAAATG	TGAGGTCAATGAAGGGGTCGT

### 2.8 Western blot

Western blot was performed to evaluate the expression of total protein, as per a previous study [16]. In brief, total protein was extracted from lung tissues by RIPA containing PMSF and protease inhibitor mixture. Protein concentration was detected by BCA protein quantification kit. Protein samples were separated by sodium dodecyl sulfate and polyacrylamide gel, then electro-transferred to the polyvinylidene difluoride membrane. The samples were blocked with 5% skim milk for 1 h at room temperature, then incubated with primary antibodies against p62 and LC3I/II at room temperature for 2 h, and the secondary antibody was labeled with HRP for 1 h at 37℃, successively. The ECL chemiluminescence solution was added for image scanning. Alpha View analysis software was used to calculate the gray value of each band. GAPDH acted as an endogenous control.

### 2.9 Statistical analysis

Data were analyzed by SPSS19.0, and expressed as mean value ± standard deviation (SD). Analysis of variance was used for comparing differences among multiple groups, least significant difference was used for pairwise comparison. *P* < 0.05 indicated a statistical significance.

## 3 Results

### 3.1 BLM dose- and time-dependently decreases arterial oxygen partial pressure in mice

**Figure 1:**
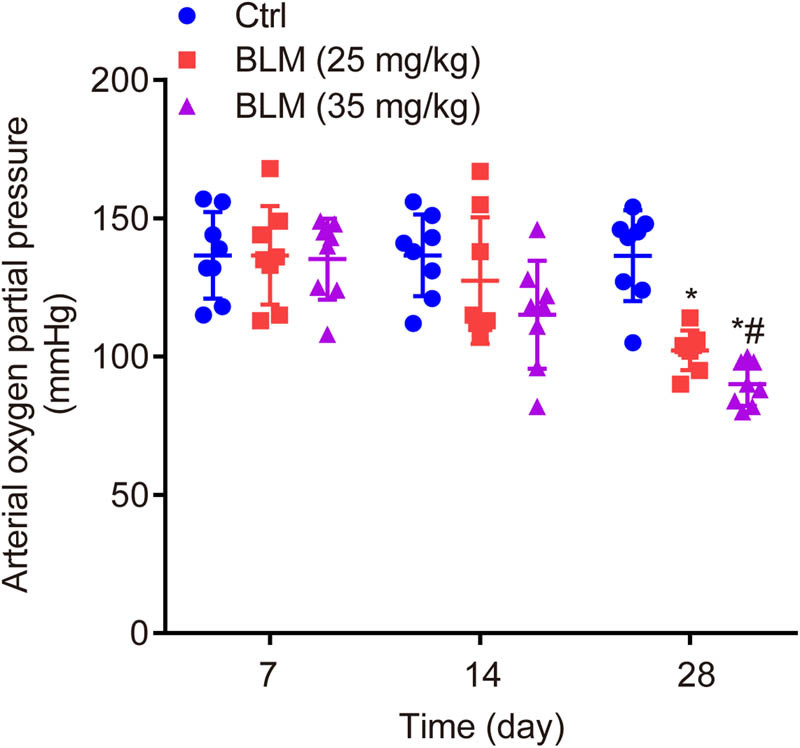
Arterial oxygen partial pressure of mice in each group. BLM dose- and time-dependently decreases arterial oxygen partial pressure. *P < 0.05 vs ctrl, ^#^ *P* < 0.05 vs BLM low group.

There was no significant difference in arterial oxygen partial pressure between ctrl and BLM groups on the 7th day (136.62 ± 17.86, 135.25 ± 14.66 vs 136.63 ± 16.45, *P* > 0.05) or 14th day (127.50 ± 22.85, 115.13 ± 19.50 vs 136.60 ± 16.32, *P* > 0.05); let alone the difference between BLM groups on the 7th day (136.62 ± 17.86 vs 135.25 ± 14.66, *P* > 0.05) or 14th day (127.50 ± 22.85 vs 115.13 ± 19.50, *P* > 0.05). Interestingly, on the 28th day, the arterial partial pressure was significantly decreased by BLM in a dose-dependent manner compared to ctrl (102.25 ± 7.19, 90.00 ± 7.86 vs 136.50 ± 16.40, *P* < 0.05, Figure 1).

Collectively, the arterial partial pressure was decreased by BLM in a dose- and time-dependent manner on the 7th, 14th, and 28th days.

### 3.2 BLM dose- and time-dependently decreases body weight, while increases wet lung weight and lung coefficient in mice

**Figure 2:**
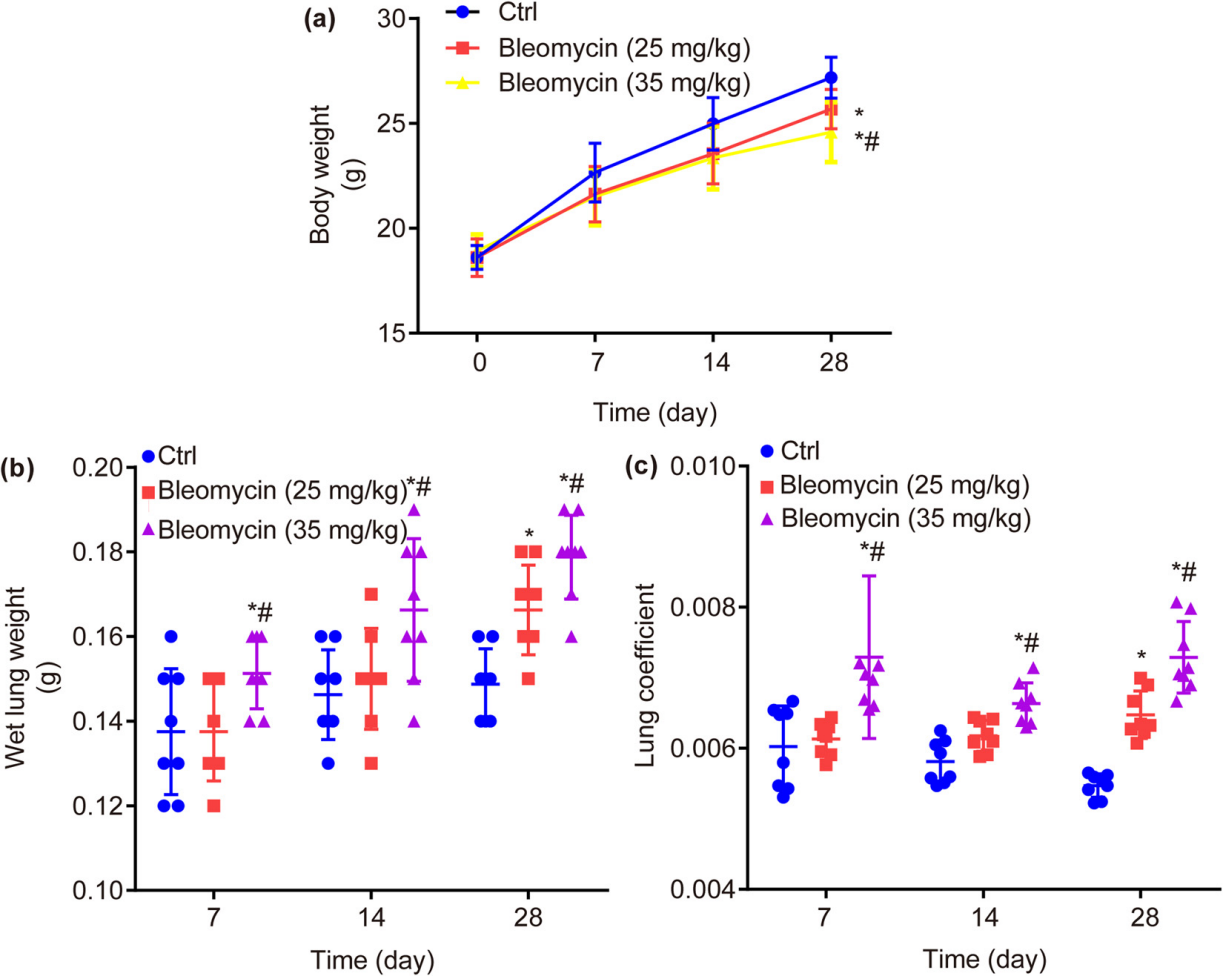
Body weight, wet lung weight, and lung coefficient of mice in each group. BLM dose- and time-dependently decreases body weight (a), while increases wet lung weight (b) and lung coefficient (c). *P < 0.05 vs ctrl, ^#^ *P* < 0.05 vs BLM low group.

There was no significant difference in body weight between ctrl and BLM groups on the 7th day (22.71 ± 0.87, 21.79 ± 1.56 vs 22.66 ± 1.40, *P* > 0.05) or 14th day (24.70 ± 0.97, 23.91 ± 1.50 vs 24.98 ± 1.25, *P* > 0.05); never mind the difference between BLM groups on the 7th day (22.71 ± 0.87 vs 21.79 ± 1.56, *P* > 0.05) or 14th day (24.70 ± 0.97 vs 23.91 ± 1.50, *P* > 0.05). Remarkably, on the 28th day, the body weight was significantly decreased by BLM in a dose-dependent manner compared with ctrl (25.68 ± 0.94, 24.58 ± 1.42 vs 27.18 ± 0.98, *P* < 0.05, Figure 2a).

In contrast to ctrl, the lung wet weight was significantly increased in BLM high group (35 mg/kg) on the 7th day (0.1513 ± 0.0084 vs 0.1376 ± 0.0086, *P* < 0.05), 14th day (0.1663 ± 0.0169 vs 0.1465 ± 0.0062, *P* < 0.05), and 28th day (0.1788 ± 0.0099 vs 0.1488 ± 0.0084, *P* < 0.05), as well as in BLM low group (25 mg/kg) on the 28th day (0.1662 ± 0.0106 vs 0.1488 ± 0.0084, *P* < 0.05) but not on the 7th day (0.1375 ± 0.0117 vs 0.1376 ± 0.0086, *P* > 0.05), or 14th day (0.1500 ± 0.0120 vs 0.1465 ± 0.0062, *P* > 0.05). Moreover, in contrast to BLM low group (25 mg/kg), the lung wet weight was significantly increased in BLM high group (35 mg/kg) on the 7th day (0.1513 ± 0.0084 vs 0.1375 ± 0.0117, *P* < 0.05), 14th day (0.1663 ± 0.0169 vs 0.1500 ± 0.0120, *P* < 0.05), and 28th day (0.1788 ± 0.0099 vs 0.1662 ± 0.0106, *P* < 0.05, Figure 2b).

In contrast to ctrl, the lung coefficient was significantly higher in BLM high group (35 mg/kg) on the 7th day (0.0073 ± 0.0012 vs 0.0053 ± 0.0002, *P* < 0.05), 14th day (0.0071 ± 0.0003 vs 0.0054 ± 0.0002, *P* < 0.05), and 28th day (0.0073 ± 0.0005 vs 0.0055 ± 0.0002, *P* < 0.05), as well as in BLM low group (25 mg/kg) on the 28th day (0.0065 ± 0.0003 vs 0.0055 ± 0.0002, *P* < 0.05) but not on the 7th day (0.0061 ± 0.0002 vs 0.0053 ± 0.0002, *P* > 0.05), or 14th day (0.0064 ± 0.0002 vs 0.0054 ± 0.0002, *P* > 0.05). Moreover, in contrast to BLM low group (25 mg/kg), the lung coefficient was significantly higher in BLM high group (35 mg/kg) on the 7th day (0.0073 ± 0.0012 vs 0.0061 ± 0.0002, *P* < 0.05), 14th day (0.0071 ± 0.0003 vs 0.0064 ± 0.0002, *P* < 0.05), and 28th day (0.0073 ± 0.0005 vs 0.0065 ± 0.0003, *P* < 0.05, Figure 2c).

Above all, the body weight (g) was decreased, while wet lung weight and lung coefficient were increased by BLM in a dose- and time-dependent manner on the 7th, 14th, and 28th days.

### 3.3 BLM dose- and time-dependently increases PF in mouse lung tissues

**Figure 3:**
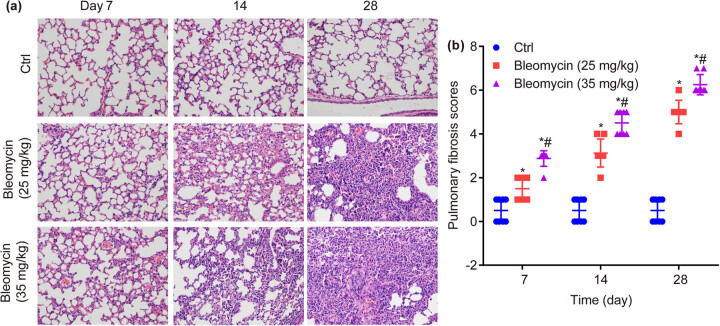
Hematoxylin–eosin (HE) staining in lung tissues of mice in each group. BLM dose- and time-dependently increases HE staining in the lower lobe of the right lung (×200) (a) and PF scores (b). *P < 0.05, vs ctrl, ^#^ *P* < 0.05 vs BLM low group.

As shown in Figure 3a, the lung tissue of alveolar walls in ctrl group was complete and the alveolar cavity structure was clear, without obvious alveolar inflammation and fibrosis. On the 7th, 14th, and 28th days, BLM dose-dependently caused damage to the surrounding subpleural lung parenchyma and bronchioles of lung tissue, structural disorder, deformation/collapse of the alveolar cavity, and focal infiltration of monocytes/lymphocytes in the lung interstitium. Specifically, on the 7th day, there was the most severe inflammation, while on the 28th day, there was the most obvious PF, part of the lung parenchyma showed large confluent consolidation and occlusion.

Subsequently, the morphological semi-quantitative score of PF was calculated. In detail, compared to ctrl, the PF score was significantly increased by BLM on the 7th day (1.50 ± 0.53, 2.88 ± 0.35 vs 0.50 ± 0.35, *P* < 0.05), 14th day (3.13 ± 0.64, 4.50 ± 0.53 vs 0.50 ± 0.46, *P* < 0.05), and 28th day (5.00 ± 0.53, 6.25 ± 0.46 vs 0.50 ± 0.53, *P* < 0.05). Furthermore, in contrast to BLM low group (25 mg/kg), the PF score was significantly increased in BLM high group (35 mg/kg) on the 7th day (2.88 ± 0.35 vs 1.50 ± 0.53, *P* < 0.05), 14th day (4.50 ± 0.53 vs 3.13 ± 0.64, *P* < 0.05), and 28th day (6.25 ± 0.46 vs 5.00 ± 0.53, *P* < 0.05, Figure 3b).

In conclusion, the PF was significantly increased by BLM in a dose- and time-dependent manner on the 7th, 14th, and 28th days.

### 3.4 BLM dose- and time-dependently increases collagen fiber deposition in mouse lung tissues

**Figure 4:**
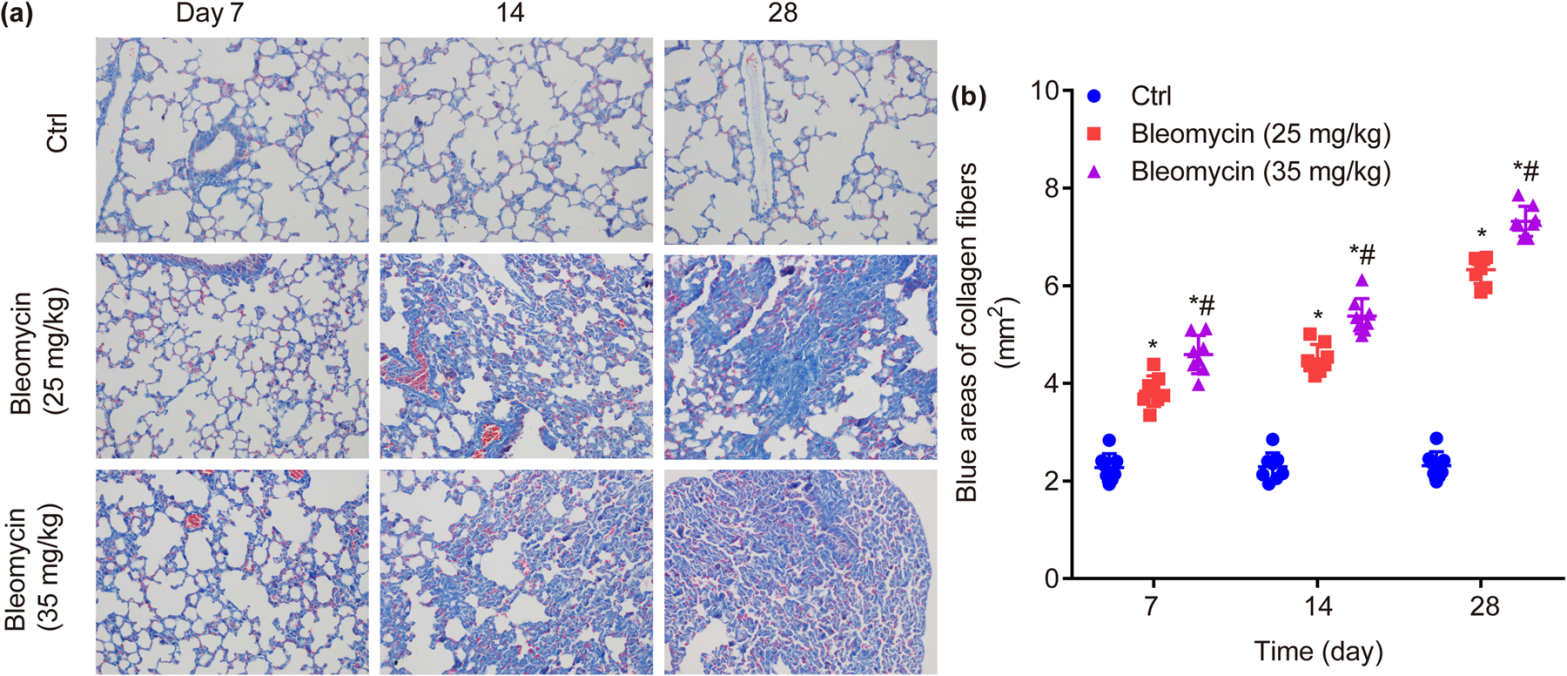
Masson staining in lung tissues of mice in each group. BLM dose- and time-dependently increases Masson staining in the lower lobe of the right lung (×200) (a) and the blue areas of collagen fibers (b). *P < 0.05 vs ctrl, ^#^ *P* < 0.05 vs BLM low group.

In ctrl group, the alveolar structure of the lung tissue was basically normal on the 7th, 14th, and 28th days; however, collagen fiber deposition was induced by BLM. In detail, on the 7th day, a small amount of collagen fiber deposition was observed in the lung tissue of BLM groups, which was mainly deposited around the blood vessels and trachea, and only a small amount of collagen fiber deposition was observed in the lung intrastromal; on the 14th day, collagen fibers mainly deposited around the walls of blood vessels, trachea, and alveoli; on the 28th day, the blue areas of collagen fibers were mainly concentrated around the trachea and blood vessels, and there were no or only a small number of punctured blue areas in the lung interstitium, the lung parenchyma formed extensive fibrosis, and the alveolar wall was thickened (Figure 4a).

In detail, compared to ctrl, the blue area of collagen fibers was significantly increased by BLM on the 7th day (3.84 ± 0.31 mm^2^, 4.59 ± 0.39 mm^2^, vs 2.28 ± 0.29 mm^2^, *P* < 0.05), 14th day (4.50 ± 0.29 mm^2^, 5.38 ± 0.36 mm^2^, vs 2.30 ± 0.27 mm^2^, *P* < 0.05), and 28th day (6.33 ± 0.28 mm^2^, 7.32 ± 0.31 mm^2^, vs 2.32 ± 0.28 mm^2^, *P* < 0.05). Furthermore, compared to BLM low group (25 mg/kg), the blue area of collagen fibers was significantly increased in BLM high group (35 mg/kg) on the 7th day (4.59 ± 0.39 mm^2^ vs 3.84 ± 0.31 mm^2^, *P* < 0.05), 14th day (5.38 ± 0.36 mm^2^ vs 4.50 ± 0.29 mm^2^, *P* < 0.05), and 28th day (7.32 ± 0.31 mm^2^ vs 6.33 ± 0.28 mm^2^, *P* < 0.05, Figure 4b).

Collectively, the collagen fiber deposition was significantly induced by BLM in a dose- and time-dependent manner on the 7th, 14th, and 28th days.

### 3.5 BLM dose- and time-dependently increases p62 protein expression, while decreases ratio of LC3II/LC3I in mouse lung tissues

**Figure 5:**
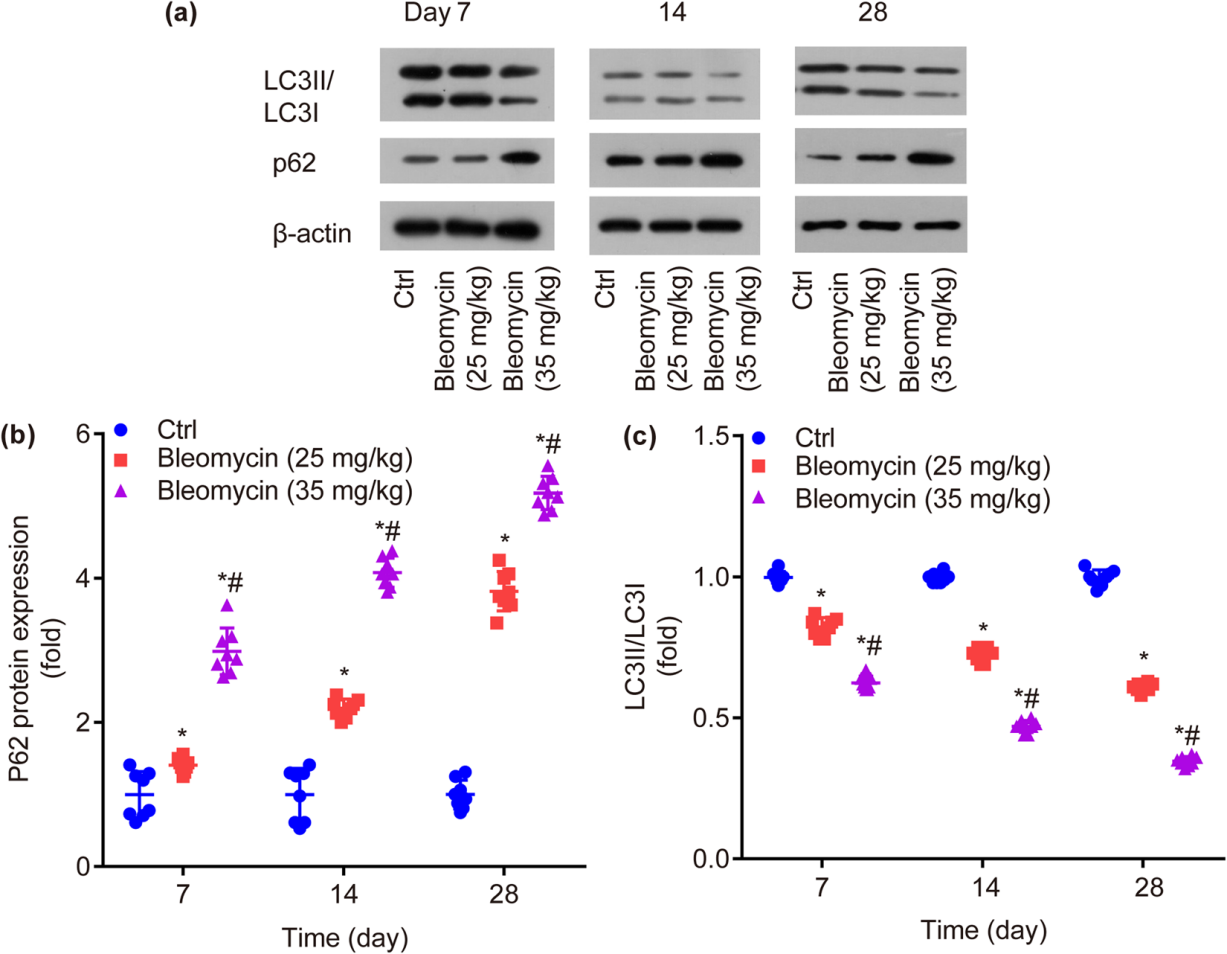
Protein expression of autophagy-related genes in lung tissue of mice in each group (a). BLM dose- and time-dependently increases p62 protein expression (b) and decreases LC3II/LC3I (c). *P < 0.05 vs ctrl, ^#^ *P* < 0.05 vs BLM low group.

Compared with ctrl, the relative expression of autophagy-related protein p62 was significantly increased by BLM in a dose- and time-dependent manner on the 7th day (*P* < 0.05), 14th day (*P* < 0.05), and 28th day (*P* < 0.05, Figure 5a and b).

Compared with ctrl, the relative expression ratio of LC3II/LC3I was significantly decreased by BLM in a dose- and time-dependent manner on the 7th day (*P* < 0.05), 14th day (*P* < 0.05), and 28th day (*P* < 0.05, Figure 5a and c).

### 3.6 BLM dose- and time-dependently decreases mRNA expressions of Atg5, Atg7, and Atg10 in mouse lung tissues

**Figure 6:**
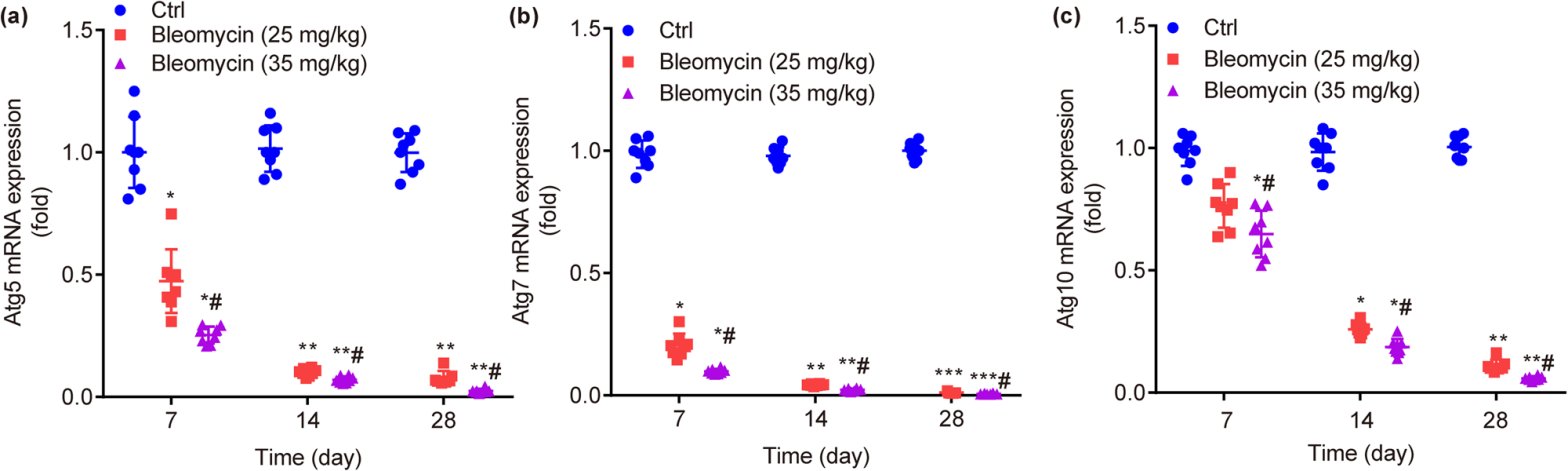
mRNA expressions of autophagy-related genes in lung tissue of mice in each group. BLM dose- and time-dependently decreases mRNA expressions of Atg5 (a), Atg7 (b), and Atg10 (c). *P < 0.05, **P < 0.01, ***P < 0.001 vs ctrl. ^#^ *P* < 0.05vs BLM low group.

Compared with ctrl, the relative mRNA expressions of autophagy-related genes Atg5 (Figure 6a), Atg7 (Figure 6b), and Atg10 (Figure 6c) were significantly decreased by BLM in a dose- and time-dependent manner on the 7th day (*P* < 0.05), 14th day (*P* < 0.05, *P* < 0.01), and 28th day (*P* < 0.01, *P* < 0.001).

## 4 Discussion

As acknowledged, chronic inflammation and certain mediators such as TNF-α lead to the formation of fibrosis and correlate with the number of inflammatory cells [17]. And modulation of apoptosis and autophagy can modulate fibrosis [18].

IPF is a group of age-related, progressive, irreversible, and fatal PF disease with unknown etiology [5]. The onset is insidious and the overall prognosis is poor [2]. Autophagy is a novel research hotspot after apoptosis, it has been widely reported in various systems and diseases. Recently, the role of autophagy in IPF has also received extensive attention [18,19]. More and more scholars believe that autophagy activity is decreased in IPF [10,20], but few reports have evaluated the relationship between autophagy and IPF. In the current study, the mouse models of IPF with different severities are constructed by inhalation of different doses of BLM, and the relative expressions of autophagy-related proteins p62, LC3II/LC3I, and autophagy-related genes Atg5, Atg7, and Atg10 in the lung tissues of the model mice are utilized to preliminarily determine the relationship between autophagy and IPF.

First, the partial pressure of oxygen and body weight were significantly lowered by BLM on the 28th day. Second, lung coefficient, lung wet weight, PF scores, and blue areas of collagen fibers were significantly elevated by BLM on the 7th, 14th, and 28th days. Additionally, the severity and distribution of the lesions in lung tissues are different, which are more consistent with the pathological changes of IPF, suggesting that the lung interstitial fibrosis model in mice constructed by atomized inhalation of BLM is closer to the IPF model. Altogether, the pulmonary interstitial fibrosis induced by aerosolized inhalation of 35 mg/kg BLM in mice is more severe than that of 25 mg/kg.

During autophagy, LC3-I (cytosolic LC3) is modified by ubiquitin like process and binds to phosphatidyl ethanolamine on the surface of the autophagy membrane to form LC3-II (membrane LC3) [21], which is then transferred to the autophagosomal precursor and autophagosomal membrane [22]. Measuring the ratio of LC3I/LC3II is widely used as detection of autophagy activity [23]. It is observed that, LC3II/LC3I ratio is significantly lowered by BLM dose- and time-dependently, which is consistent with that reported by Zhu et al., who find that LC3II/LC3I ratio is reduced in PF lung tissue [24].

p62 protein migrates to the place of autophagosome, then it binds to LC3 and ubiquitin-degraded protein [25]. p62 is deemed to be the receptor for ubiquitin-degraded substrates which forms autophagosome [26]. When autophagy activity is inhibited or defective, p62 and ubiquitinated protein accumulate in a large number of cells, leading to a series of pathophysiological disorders and eventually the occurrence of diseases [27,28]. It is found that, p62 protein is significantly increased by BLM dose- and time-dependently, which is consistent with Zeng et al., who found the increased expression of p62 protein in lung tissues of PF models by monitoring autophagy activity markers [29].

As acknowledged, knockdown of Atg5 results in decline or complete inhibition of autophagy [30]. Atg7, which functions as a ubiquitination activase, encodes a ubiquitin E1-like enzyme that activates the ubiquitin-like protein Atg12 and all Atg8 in mammals, and mouse endothelial cells are susceptible to BLM-induced PF after knockout of Atg7 [31]. Atgl0, which acts as a ubiquitin cross-linking enzyme (E2-like enzyme), together with Atg7, is involved in the formation of ATGL2-ATG5 ubiquitination complex, thus promoting the formation/extension of autophagosome membrane [32,33]. It is discovered that, the relative expression levels of autophagy-related genes Atg5, Atg7, and Atg10 are significantly lowered by BLM dose- and time-dependently.

Altogether, current study preliminarily demonstrates that the more severe the BLM-induced PF in mice, the lower the autophagy activity.
